# Comparison of the 3D triple-scan protocol and the impression replica technique for 3-unit tooth-supported fixed dental prostheses

**DOI:** 10.1080/26415275.2019.1684198

**Published:** 2019-10-30

**Authors:** Per Svanborg, Miranda Andersson, Therese Reinedahl, Torgny Alstad

**Affiliations:** Department of Prosthodontics/Dental Materials Science, Institute of Odontology, the Sahlgrenska Academy, University of Gothenburg, Göteborg, Sweden

**Keywords:** Dental marginal adaptation, dental prosthesis, dental impression technique, restoration fit evaluation

## Abstract

**Objective:** The aim of this study was to compare the fit measurements from the 3 D triple-scan protocol and the impression replica technique, by measuring the fit of three-unit tooth-supported fixed dental prostheses.

**Materials and methods:** The results from an earlier study using the triple-scan protocol were compared to results from the impression replica technique for absolute marginal gap, cervical area gap and internal gap.

**Results: **There were no differences for cervical area gap or internal gap, but for absolute marginal gap the impression replica technique obtained significantly smaller fit values.

**Conclusion:** The impression replica technique and the triple-scan protocol may both be used to measure the fit of tooth-supported restorations. However, the scanner used for the triple-scan protocol must be able to obtain scan points at the outermost edge of the restorations when used for absolute marginal gap.

## Introduction

A factor that may be important for the longevity of tooth-supported fixed dental prostheses (FDP) is the fit, the ‘misfit’ measured at various points between the tooth and the restoration [1]. The fit of tooth-supported restorations has been evaluated using the impression replica technique since the 1970s. The technique creates a silicone replica of the tooth-crown intaglio and is most often used to analyze two-dimensional (2 D) measurements using slices of the replica, which may not represent the overall fit of the crown. However, it can be used both *in vivo* and *in vitro*. Holst et al. proposed a new technique, where the total area of tooth-restoration intaglio could be measured and visualized [2]. The triple-scan protocol is a technique where an optical scanner is used to scan the model, the inside of the restoration and the restoration placed on the model. The scans are then imported into a CAD software program where the distances between model/teeth and the intaglio of the restoration can be measured [2,3].

### Aim

To compare the fit measurements from the 3 D triple-scan protocol and the impression replica technique, by measuring the fit of three-unit tooth-supported fixed dental prostheses.

## Material and methods

**Figure 1.**
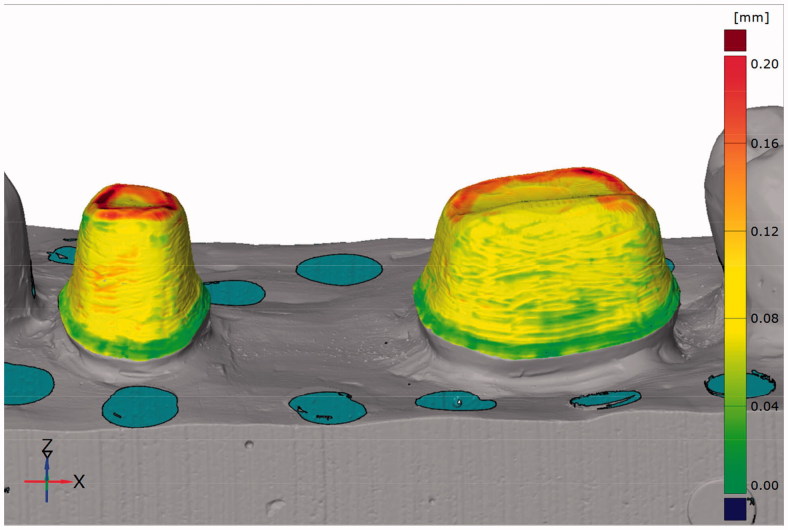
Image from the CAD software used for fit measurements with the triple-scan protocol. Measuring points are obtained from the full surface of the tooth-restoration intaglio.

**Figure 2.**
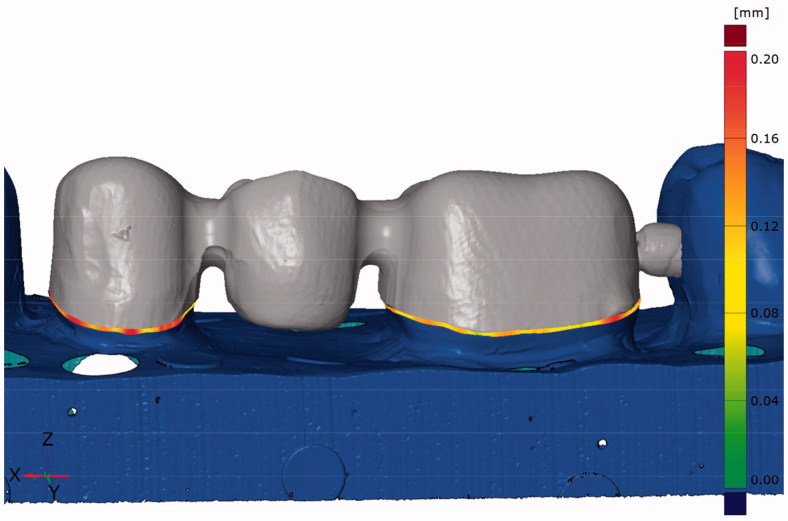
Image from the CAD software used for fit measurements with the triple-scan protocol. The measurements for the absolute marginal gap are indicated at the preparation margin.

Ten models of a mandibular arch with prepared teeth (34^35^36) for a three-unit FDP were scanned using an iTero digital impression scanner and one CNC-milled CoCr FDP (Straumann Coron) for each model were fabricated and measured for fit using the triple-scan protocol as reported in an earlier publication (Figures 1 and 2) [3]. For this study, the fit was measured using the impression replica technique and thereafter the results were compared.

**Figure 3.**
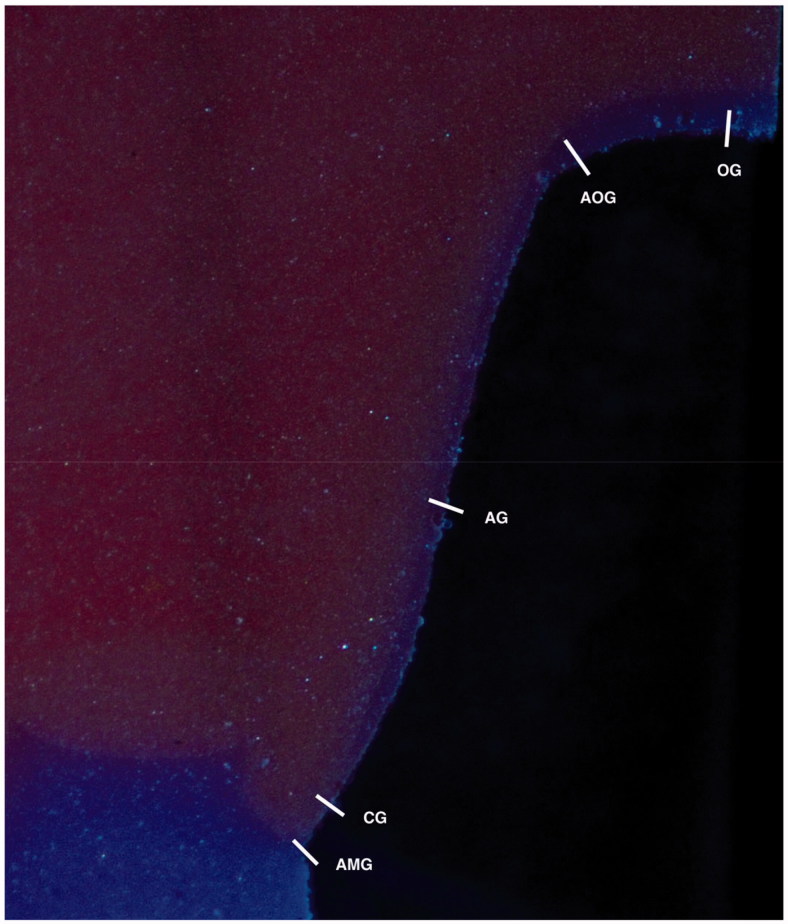
Image of impression replica from the microscopic measurements with measuring areas. AMG: Absolute marginal gap; CG: Cervical area gap; AG: Axial gap; AOG: Axial-occlusal gap; OG: Occlusal gap.

The FDPs were filled with a light body impression material (Elite HD+, Super Light Fast, Zhermack SpA, Badia Polesine, Italy) and placed on the models using a constant finger pressure for three minutes. After another three minutes, the FDPs were carefully removed and the light body material remained on the model. Impression trays were filled with heavy body impression material (Elite HD+, Tray Heavy Body, Zhermack SpA, Badia Polesine, Italy) and placed on the model. The impression trays were removed after ten minutes. The silicone replicas were cut with a razor blade in a standardized manner, the premolars once buccolingually and once mesial-distally, the molars were cut once mesial-distally and twice buccolingually according to Reich et al. [4]. A stereomicroscope (Nikon SMZ800, Zoom Stereomicroscope, Tokyo, Japan) with x30 magnification was used to measure the gap in five areas; absolute marginal gap, cervical area gap, axial gap, axial-occlusal gap and occlusal gap (Figure 3). In total, 500 measurement points were collected, 50 per FDP (20 points for the premolar and 30 for the molar). The cervical area gap, axial gap, axial-occlusal gap and occlusal gap were used to calculate a total mean of the internal fit named internal gap.

### Statistics

The values of fit for absolute marginal gap, cervical area gap and internal gap from the impression replica technique and the triple scan protocol were compared using the related samples Wilcoxon signed ranks test. The significance level was set at 5%.

## Results

The results showed no statistical significant differences for cervical area gap or internal gap between the two techniques. However, there was a statistically significant difference for absolute marginal gap, premolar *p* = .005, molar *p* = .047, and FDP *p* = .005, with a smaller measured gap for the impression replica technique (Table 1).

**Table 1.** Comparison of fit values (in μm) for the triple scan protocol and the impression replica technique.

Measuring technique		AMG		CG		IG	
	Teeth	Mean	SD	Mean	SD	Mean	SD
Replica	PM	60	12	46	7	87	8
Triple scan	PM	146*	45	44	12	91	9
Replica	M	118	26	49	7	104	15
Triple scan	M	139*	25	44	6	95	9
Replica	FDP	94	18	48	6	97	11
Triple scan	FDP	142*	33	44	8	93	8

PM: premolar; M: molar; FDP: 34–36; AMG: Absolute marginal gap; CG: Cervical area gap; IG: Internal gap; SD: Standard deviation.

*Statistically significant difference between Replica and Triple scan using the related samples Wilcoxon signed ranks test.

## Discussion

The results show that the impression replica technique using only slices of the 2 D fit may be as accurate as the triple-scan protocol for internal fit measurements. Other studies have evaluated the impression replica technique for validity and reliability and have concluded that it has a tendency to overestimate the gap with 2 to 11% and have moderate variations in reliability [5,6]. For cervical area gap and internal gap, the impression replica technique had slightly higher values but these differences were not statistically significant. The pre-set spacer was set at 30 μm from the margin and 0.5 mm axially, and 60 μm above. With both measuring techniques, the mean of the internal gap was 97 and 93 μm respectively.

The advantages of the impression replica technique are that it can be used both *in vitro* and in vivo, and it does not require expensive equipment. However, the limitations are possible ruptures in the light-body silicone when removing the restoration and uncertainties regarding the placement and direction of the sections [7]. With the triple-scan protocol, the intaglio of the tooth and inner surface of the restoration can be visualized and measured but sections or slices may also be isolated from the data [8].

Interestingly, the only significant differences were found for absolute marginal gap where the replica technique showed a smaller gap compared to the triple-scan protocol. This could be attributed to the scanner used and its capacity to scan the outermost edge of the restorations, or the analysis technique used to measure the absolute marginal gap. The scanner records measurement points at an interval and subsequently connects the points, the larger the interval the greater the risk of the last point being imprecise in relation to the sharp edge of the restoration. The results obtained from the replica technique are lower and could be explained by a more accurate technique to record the true value for AMG or possible ruptures in the silicone replica. However, ruptures would have been detected during the microscope analysis. Another aspect of the impression replica technique is the pressure applied to the FDP when recording the cement space. If the pressure was larger when using the impression replica technique compared to the triple-scan protocol it could result in a tighter fit at the margin. Although this should also have resulted in a smaller internal fit for the impression replica technique, the results showed a slightly larger internal gap, 97 μm compared to 93 μm for the triple-scan protocol.

### Conclusion

The impression replica technique and the triple-scan protocol may both be used to measure the fit of tooth-supported restorations. However, the scanner used for the triple-scan protocol must be able to obtain scan points at the outermost edge of the restorations when used for absolute marginal gap.

## Acknowledgements

The authors wish to thank Sebastian Stolze for statistical assistance and Petra Hammarström Johansson for instructions and guidance regarding the microscope.
